# Breast cancers utilize hypoxic glycogen stores via PYGB, the brain isoform of glycogen phosphorylase, to promote metastatic phenotypes

**DOI:** 10.1371/journal.pone.0220973

**Published:** 2019-09-19

**Authors:** Megan A. Altemus, Laura E. Goo, Andrew C. Little, Joel A. Yates, Hannah G. Cheriyan, Zhi Fen Wu, Sofia D. Merajver

**Affiliations:** 1 Graduate Program in Cancer Biology, University of Michigan, Ann Arbor, Michigan, United States of America; 2 Department of Internal Medicine, University of Michigan, Ann Arbor, Michigan, United States of America; 3 Rogel Cancer Center, University of Michigan, Ann Arbor, Michigan, United States of America; Wayne State University, UNITED STATES

## Abstract

In breast cancer, tumor hypoxia has been linked to poor prognosis and increased metastasis. Hypoxia activates transcriptional programs in cancer cells that lead to increased motility and invasion, as well as various metabolic changes. One of these metabolic changes, an increase in glycogen metabolism, has been further associated with protection from reactive oxygen species damage that may lead to premature senescence. Here we report that breast cancer cells significantly increase glycogen stores in response to hypoxia. We found that knockdown of the brain isoform of an enzyme that catalyzes glycogen breakdown, glycogen phosphorylase B (PYGB), but not the liver isoform, PYGL, inhibited glycogen utilization in estrogen receptor negative and positive breast cancer cells; whereas both independently inhibited glycogen utilization in the normal-like breast epithelial cell line MCF-10A. Functionally, PYGB knockdown and the resulting inhibition of glycogen utilization resulted in significantly decreased wound-healing capability in MCF-7 cells and a decrease in invasive potential of MDA-MB-231 cells. Thus, we identify PYGB as a novel metabolic target with potential applications in the management and/or prevention of metastasis in breast cancer.

## Introduction

More than half of solid tumors present with locally hypoxic or anoxic areas relative to the surrounding normal tissue [1]. Hypoxia has been associated with metastasis and poor prognosis in many cancers, including breast [1,2]. Tumor hypoxia results from an imbalance between oxygen delivered to the tumor niche and its consumption by cancer cells and tumor associated cells. Hypoxia develops in primary solid tumors due to multiple factors, including increased distance from blood supply, weakened vessel integrity, and competition for oxygen and nutrients from neighboring tumor and tumor-associated cells [1]. Both tumor and normal cells respond to a hypoxic environment by activating specific signaling pathways that lead to distinct gene expression changes, amongst the most immediate and salient common hubs being stabilization of the hypoxia-inducible factors, HIF-1α and HIF-2α [3].

In cancer, stabilized HIF-1α activates transcriptional programs that have been recognized to induce the epithelial to mesenchymal transition (EMT) and support metastasis in various cancer types [4–7]. Hypoxia-induced transcription factors and signaling pathways include Twist, Snail, ZEB1, Notch, TGF-β, and Hedgehog, among others[6,8–14]. Moreover, HIF1-α and hypoxia have been shown to increase the metastatic phenotype in multiple cancer cell types, including in breast cancer *in vivo* experiments, and have been linked to increased risk of metastasis and mortality in breast cancer patient cohorts [7,15–22]. Investigation of how the hypoxic tumor microenvironment contributes to increased cancer aggressiveness and metastatic potential may provide novel therapeutic avenues.

Hypoxia and subsequent stabilization of HIF-1α induce downstream metabolic changes in cancer cells. These include increased expression of glucose transporters and genes involved in glycolysis, altered fatty-acid and lipid metabolism, and increased pyruvate dehydrogenase kinase activity thereby decreasing the amount of pyruvate that enters the TCA cycle and decreasing oxidative phosphorylation [23–31]. One additional metabolic change is glycogen accumulation, which has been previously described in both cancerous and non-cancerous cells [32–35] under hypoxic conditions relative to their normal state. A variety of methods have been employed to exploit the potential vulnerabilities that arise from tumors’ adaptations to hypoxia, and have been shown to contribute to tumor control [36]. In this study, we focus on a deeper understanding of the potential vulnerabilities exhibited by the hypoxic modulation of glycogen homeostasis, carried out by a delicate balance between synthesizers and degraders of glycogen.

Glycogen is a high molecular weight branched polysaccharide of glucose and is the main glucose storage macromolecule in animals [37]. It is primarily stored in the liver where it is utilized to maintain blood-glucose levels and in the muscles where it can be mobilized quickly for energy production during exercise [37]. Glycogen is synthesized around a glycogenin core by addition of UDP-glucose onto growing glycogen chains. Glucose-1-phosphate available in the cell from either glucose transported into the cell or gluconeogenic substrates is catalyzed to UDP-glucose by UDP-glucose pyrophosphorylase-2 (UGP2). UDP-glucose is added onto glycogen via an α-1,4 linkage by glycogen synthase, the rate limiting enzyme in glycogen synthesis [37]. There are two isoforms of glycogen synthase: GYS1 which is mainly expressed in the liver and the muscle isoform GYS2. During degradation, glucose-1-phosphate molecules are removed from the non-reducing end of the glycogen molecule by glycogen phosphorylase [37] (PYG), the rate limiting enzyme of glycogen degradation. PYG has three different isoforms in humans that are typically expressed in different tissues: liver (PYGL), muscle (PYGM), and brain isoforms (PYGB). Free glucose-1-phosphate molecules are then catalyzed to glucose-6-phosphate, the first intermediate in glycolysis, by phosphoglucomutase-1 (PGM1).

To maintain glycogen-free glucose balance, glycogen synthase and glycogen phosphorylase are tightly regulated. Glycogen synthase activity is regulated allosterically and via posttranslational modification e.g. phsphorylation. Phosphorylation of glycogen synthase by glycogen synthase kinase 3α and 3β (GSK3α/β) at multiple serine residues inhibits its activity [38]. Glycogen phosphorylase is activated by phosphorylation at Ser-14 by phosphorylase kinase [37] and by the allosteric stimulator glucose-6-phosphate (G6P). Importantly, both glycogen synthase and glycogen phosphorylase are regulated in synchrony by protein phosphatase-1 (PP1). PP1 dephosphorylation activates glycogen synthase, but inhibits glycogen phosphorylase, leading to reciprocal regulation of glycogen synthesis and degradation [37].

High levels of glycogen have been found in diverse cancer cell types including breast cancers [39]. Recently, glycogen levels were found to be inversely correlated with proliferation rate, indicating that glycogen was utilized as an energy source to sustain proliferation[39]. High levels of glycogen have also been found in the hypoxic tumor cores and in tumors treated with anti-angiogenic therapies [40]. Hypoxia and stabilization of HIF-1α have been shown to increase levels of many glycogen enzymes and regulatory proteins including UGP2, GYS1, GBE, and PPP1R3C, the glycogen-associated regulatory subunit 3C of PP1 [35,41–43]. Additionally, in work conducted by Favaro et al., siRNA knockdown of the liver isoform of glycogen phosphorylase was shown to inhibit glioblastoma cell proliferation under hypoxia and induce senescence in a reactive oxygen species-dependent manner [40]. While past studies have focused on glycogen accumulation as protection from the adverse hypoxic environment at the primary tumor site, our work aims to determine the relationship between glycogen accumulation and energy reserves utilized for hypoxia driven metastasis.

In this study, we sought to understand the link between the fuel provided by hypoxia-induced glycogen storage in aggressive breast cancers and the promotion of invasion and migration. We found that six different breast cancer cell lines and a normal-like breast epithelial cell line all increased their glycogen stores under hypoxia. Glycogen gene expression changes under hypoxia were also evaluated, finding no consensus change in expression that would account for this increase, indicating other means of regulation of glycogen stores are in place, such as post-translational modification or allosteric regulation of the rate-limiting enzymes. In order to investigate how proliferation, migration, and invasion are affected by glycogen storage and utilization, we created glycogen phosphorylase knockdowns for both the liver and brain isoforms of PYG. In the two breast cancer cell lines, MDA-MB-231 and MCF-7, loss of the brain isoform PYGB inhibited hypoxic glycogen usage whereas the loss of both PYGL and PYGB in the normal-like MCF-10A cell line exhibited this effect. Prohibition of glycogen utilization resulted in a marked decrease of proliferation in MCF-10A cells and a slight decrease in MCF-7 cells. Wound-healing was strikingly decreased in shPYGB MCF-7 cells under both normoxic and hypoxic conditions. While loss of PYGB did not affect the proliferation or wound-healing of triple-negative breast cancer (TNBC) MDA-MB-231 cells, it did significantly decrease the invasiveness of these cells. These findings indicate that attacking the cancer vulnerabilities derived from dysregulation of glycogen metabolism could be a therapeutic strategy, not only to slow tumor growth as has been previously suggested by other work, but also to inhibit development of distant metastases in breast cancers such as TNBC, for which few targeted therapies currently exist.

## Materials and methods

### Cell culture and media

MDA-MB-231 cells (ATCC HTB-26) were maintained in RPMI-1640 (+) L-glutamine (ThermoFisher 11875093) supplemented with 10% fetal bovine serum (FBS) (Corning 35-010-CV). SUM-149 [44] cells (gift from Dr. Steve Ethier, Medical University of South Carolina) were maintained in Ham’s F-12 (+) L-glutamine (ThermoFisher 11765054) supplemented with 5% FBS (GE Healthcare Life Sciences SH30071.03), 5μg/mL insulin, and 1μg/mL hydrocortisone. MCF-7 and MDA-468 (ATCC HTB-132) cells were maintained in DMEM (Corning 10-013-CV) supplemented with 10% FBS (Corning 35-010-CV). MCF-10A cells (ATCC CRL-10317) were maintained in 50:50 DMEM/F-12 (Corning 10-090-CV) supplemented with 5% horse serum (ATCC 30–2040), 10 μg/mL insulin, 0.5 μg/mL hydrocortisone, 0.02μg/mL epidermal growth factor, and 0.1 μg/mL cholera toxin. BT-549 cells (ATCC HTB-122) were maintained in RPMI-1640 (+) L-glutamine (ThermoFisher 11875093) supplemented with 10% FBS (Corning 35-010-CV) and 0.8 μg/mL insulin. SUM-190 [45] cells (gift from Dr. Steve Ethier, Medical University of South Carolina) were maintained in Ham’s F-12 (+) L-glutamine (ThermoFisher 11765054) supplemented with 1mg/mL bovine serum albumin (BSA) (Sigma-Aldrich A8806), 1X ITS-X (ThermoFisher 51500056), 10 nM T3, 10 mM HEPES, and 1μg/mL hydrocortisone. All cell lines were supplemented with 1X Anti-Anti (ThermoFisher 15240062) and 5 μg/mL gentamicin (ThermoFisher 15750060), except SUM-149 and SUM-190 which were supplemented with 2.5μg/mL amphotericin B (ThermoFisher 15290018), 50 U/mL penicillin-streptomycin, and 5 μg/mL gentamicin (ThermoFisher 15750060). Metabolism media (MM) was made from base DMEM (Sigma-Aldrich D5030) which was supplemented with 44 mM sodium bicarbonate, 11 mM glucose, 2.5 mM glutamine, and 1 mM sodium pyruvate as well as all cell-line specific supplements listed above. All cells were grown in 5% CO_2,_ except SUM-149 and SUM-190 which were grown at 10% CO_2_. All hypoxia experiments were performed at 1% O_2_ with stated CO_2_ concentration in an incubator system with O_2_ and CO_2_ sensors to maintain accurate gas concentration.

### Glycogen assay

For each experiment four 10-cm plates of cells were plated in normal growth media and grown in CO_2_ controlled normal atmosphere (normoxia) for 24hr. Plates were washed with Dulbecco’s phosphate-buffered saline (DPBS) (ThermoFisher 14190250) and changed to MM for one hour. Media was changed again with MM and two plates were placed at 1% O_2_ and two were kept in normoxic conditions. After 24h and 48h, cells from one plate from each condition were harvested with 0.05% trypsin (ThermoFisher 25300054) and pelleted via centrifugation. Cell pellets were washed with DPBS and centrifuged a second time. Cell pellet was flash frozen in liquid nitrogen and stored at -80°C for up to three days. Glycogen assay was conducted using a glycogen assay kit (Cayman Chemical 700480). Cell pellets were re-suspended in 400mL Assay Buffer and homogenized using 1mL pestle tissue grinder. Glycogen concentration was normalized to protein concentration in lysate. Each glycogen assay was ran on three separate dates in biological triplicates, and each experiment on a given date performed in technical triplicate, for a total of 9 (3x3) replicates. Averages were calculated by first averaging the technical replicates for each date then averaging those three values and taking the standard error between biological triplicate. One-way ANOVA multiple comparison statistics were calculated using GraphPad Prism 7.04 software. Glycogen utilization assays for knockdown cell lines were conducted as described above with the following alterations: two plates of each cell line were placed in 1% O_2_ conditions for 24hr, following which one plate was harvested and the second plate was moved to normoxic conditions for 24hr before harvesting.

### Periodic acid-Schiff staining

Cells were grown in 6-well tissue culture plate on acid-washed coverslips. Coverslips were prepared by heating overnight in 1M HCl at 55°C. Solution was cooled to room temperature and coverslips were rinsed in ddH_2_O. Coverslips were then rinsed in a sonicating water bath in fresh ddH_2_O for 15min 3X, followed by 50%, 70%, then 95% ethanol for 15min each. Coverslips were stored in 95% ethanol. Once cells reached 50% confluent media was changed to MM and cells were incubated in normoxia or 1% O_2_ conditions for 48hr. Cells were fixed in Carnoy’s solution of 60% ethanol, 30% chloroform, and 10% glacial acetic acid, permeabilized in 0.5% Triton X-100 at 4°C and quenched in PBS-glycine. Amylase controls were digested in 0.05mg/mL α-amylase (Sigma-Aldrich A3176) at room temperature. All coverslips were incubated in Periodic Acid Solution (Sigma-Aldrich 395B) and then Schiff’s Reagent (Sigma-Aldrich 395B). Nuclei were counterstained in Hematoxylin Gill’s Solution No. 3 (Sigma-Aldrich 395B) and blued in Scott’s Tap Water Substitute (3.5 g sodium bicarbonate, 20 g magnesium sulfate, 1L distilled H_2_O). Coverslips were air dried and mounted on slides for imaging.

### RT-qPCR

RNA was isolated for all cells using RNeasy mini kit (Qiagen) and reverse transcription for cDNA preparation was performed using Reverse Transcription System (Promega) according to manufacturer’s instructions. qPCR was performed using QuantiTect SYBR Green PCR kit (Qiagen) on a Step-One Plus real-time PCR system (Applied Biosystems). Each qPCR analysis was performed with five technical replicates and on three separate dates in biological triplicate for a total of 15 (3x5) replicates. Averages were calculated by first averaging the technical replicates for each date then averaging those three values and taking the standard error between biological repeats. Ct values were normalized to RPL22 and RPL30. Statistical analysis for each triplicate was performed on dCT values using multiple comparisons one-way ANOVA in Graphpad Prism 7.04. qPCR primers utilized in study are reported in S1 Table.

### shRNA knockdown

Annealed shRNA oligonucleotides were cloned into pLentilox 3.7 GFP lentiviral expression vector obtained from University of Michigan Vector Core using restriction enzymes XhoI and HpaI. shRNA expression is driven by mU6 promoter with selection marker GFP expression driven by CMV promoter. An empty vector (EV) pLentilox 3.7 GFP plasmid was used as a control. Lentivirus was produced by University of Michigan Vector Core. After viral transduction all cell lines were sorted for GFP expression and viability using the University of Michigan Flow Cytometry Core. shRNA oligonucleotides utilized are detailed in S2 Table. Knockdowns were confirmed via western blot and RT-qPCR.

### Western blotting

Protein samples were harvested from 6cm tissue culture dish using RIPA buffer with protease inhibitors (cOmplete Mini Protease Inhibitor Cocktail, Sigma 11836153001). Protein concentration in lysate determined using BCA protein assay (ThermoFisher 23225). 40 μg protein per sample were loaded onto pre-cast 4–12% Tris-HCL gel (Bio Rad 3450027). Gel was transferred onto nitrocellulose membrane (ThermorFisher IB23001) using iBlot2 system (ThermoFisher IB21001) and blocked in 5% blotting-grade non-fat dry milk (Bio Rad 1706404). Primary antibodies utilized were PYGL rabbit polyclonal antibody (ThermoFisher PA5-51492), GPBB (PYGB) rabbit polyclonal antibody (ThermoFisher PA5-28022), and Actin mouse monoclonal antibody (Sigma A3854). Un-cropped images of western blots are reported in S1–S4 Images.

### Proliferation assay

Cells were incubated for 24hr at normoxic and 1% O_2_ conditions. After 24hr cells were plated in black clear-bottom 96-well plates in technical quintuplet at 2,000 cells/well for MDA-MB-231 and 3,000 cells/well for MCF-7 and MCF-10A and incubated overnight at normoxia or 1% O_2_ conditions. Media was changed and bright-field images were taken every four hours for five days using Biotek Cytation5 and oxygen controlled Biotek Biospa8 system. Experiments were performed using five technical replicates and on three separate dates in biological triplicate for a total of 15 (3x5) replicates. Cell counts were calculated at each timepoint using Biotek Gen5 software. Cell count data for each technical replicate over time were fit to exponential curves using GraphPad Prism 7.04. Technical replicates were discarded if curve fit R^2^ value was less than 0.95 except for MCF10A which were discarded if value was less than 0.55 due to the very slow proliferation of shPYG cell lines under hypoxic conditions affecting the exponential curve fit. Geometric mean and geometric standard deviation of technical replicate rate constants were calculated for each biological replicate. Multiple comparisons one-way ANOVA were conducted on rate constants of three biological replicates to determine significance.

### Wound-healing assay

Cells were incubated in MM (see *cell culture and media*) for 24hr at normoxia and 1% O_2_ conditions. After 24hr cells were plated in Ibidi 2-well culture inserts (Ibidi 80209) affixed to 12-well plates at 50,000 cells/well in technical triplicate and incubated overnight at normoxia and 1% O_2_ conditions. After overnight incubation, inserts were removed and washed 2X with PBS before adding fresh MM. Bright-field images were taken every four hours until wounds were fully closed using Biotek Cytation5 and oxygen controlled Biospa8 system. Wound area for each image in pixels was calculated using the ImageJ macro MRI Wound Healing Tool. These values were used to calculate the percent closure of the wound at each time point. Experiments were performed in technical triplicate and on three separate dates in biological triplicate for a total of 9 (3x3) replicates. Reported values are the average percent closure and standard error of biological triplicates at each timepoint. p-values were determined using two-way ANOVA in GraphPad Prism 7.04.

### Transwell invasion assay

Cells were incubated in MM for 24hr at normoxia and 1% O_2_ conditions. After 24hr cells were plated in Corning BioCoat Matrigel Invasion Chambers (Corning 354480) at 100,000 cells/chamber in serum-free MM with serum-containing MM in bottom chamber in technical duplicate. Cells were allowed to invade for 24hr in normoxia or 1% O_2_ conditions, respectively. After invasion, Matrigel and top chamber were scrubbed to remove non-invading cells. Inserts were fixed in 70% ethanol and stained in 0.2% Crystal Violet. Bottom of insert was removed and mounted on slides for imaging. 5 images per insert were taken at 10X magnification. Each image was processed using ImageJ. Images were converted to binary in ImageJ and converted to percent coverage based on total pixels per image. Statistical analyses were conducted on biological triplicates, which were the averages of each of the technical duplicate, which in turn was the average of five fields of vision, for a total of 6 replicates and a corresponding thirty fields of vision, using multiple comparisons one-way ANOVA in GraphPad Prism 7.04.

## Results

**Fig 1:**
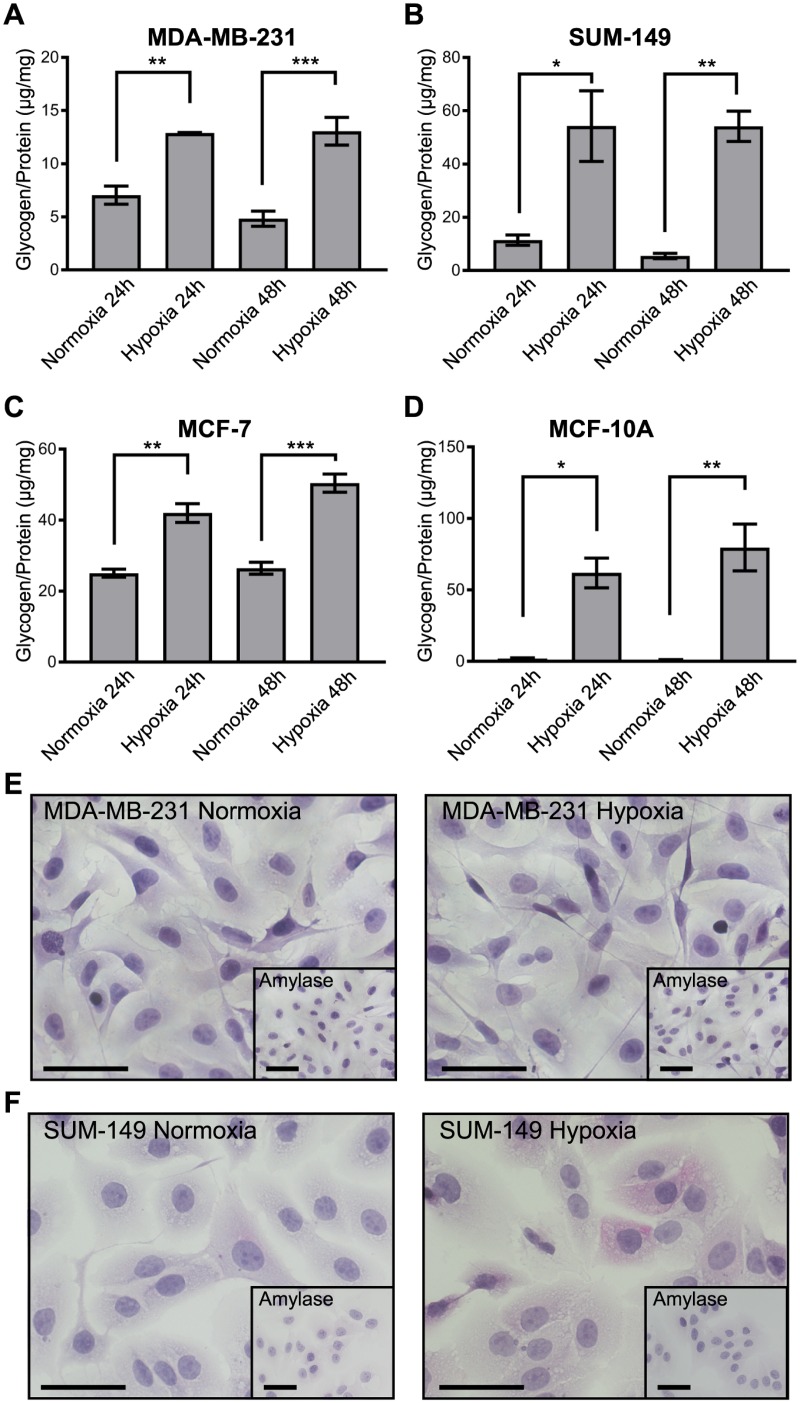
Glycogen accumulates in breast cancer cells under hypoxic conditions across subtypes. Glycogen levels normalized to total protein lysate in (A) MDA-MB-231, (B) SUM-149, (C) MCF-7, and (D) MCF-10A cell lines at 24 and 48h after media change and exposure to normoxia or 1% O_2_ (hypoxia) conditions. Error bars equal SEM of n = 3. *p<0.05, **p<0.01, ***p<0.001 by multiple comparisons one-way ANOVA.

Glycogen levels were quantified in six different breast cancer cell lines and a normal-like breast cell line at 24 and 48h exposure to normoxia and 1% O_2_ hypoxia (Fig 1A–1D and S1 Fig). Glycogen levels were significantly increased in the TNBC cell line MDA-MB-231, inflammatory TNBC SUM-149, ER+ MCF-7, and normal-like MCF-10A (Fig 1). However, there was great variation in the extent to which glycogen accumulation increased under hypoxia compared to normoxia. MDA-MB-231 and MCF-7 cells’ glycogen levels increased 1.5-3X under hypoxia, while SUM-149 and MCF-10A increased from 10-75X. In addition, we find variable basal glycogen levels in normoxia, with MCF-7 having the highest normoxic glycogen levels and normal-like MCF-10A having essentially no normoxic glycogen stores. Periodic-acid Schiff (PAS) staining for glycogen was conducted on MDA-MB-231 and SUM-149 cell lines to visually confirm glycogen accumulation (S1D and S1E Fig). Glycogen accumulation was observed via PAS staining with no detectable change in staining intensity between normoxic and hypoxic MDA-MB-231. SUM-149, with higher hypoxic glycogen increases, as determined via the glycogen assay (Fig 1B), exhibited a diffuse cytoplasmic pink staining in hypoxic, but not normoxic conditions. These results, paired with data from additional TNBC cell lines MDA-MB-468, BT-549, and the inflammatory HER-2+ breast cancer cell line SUM-190 (S1A and S1B Fig), show a wide range of normoxic glycogen storage and glycogen accumulation in response to hypoxia in all the subtypes.

**Fig 2:**
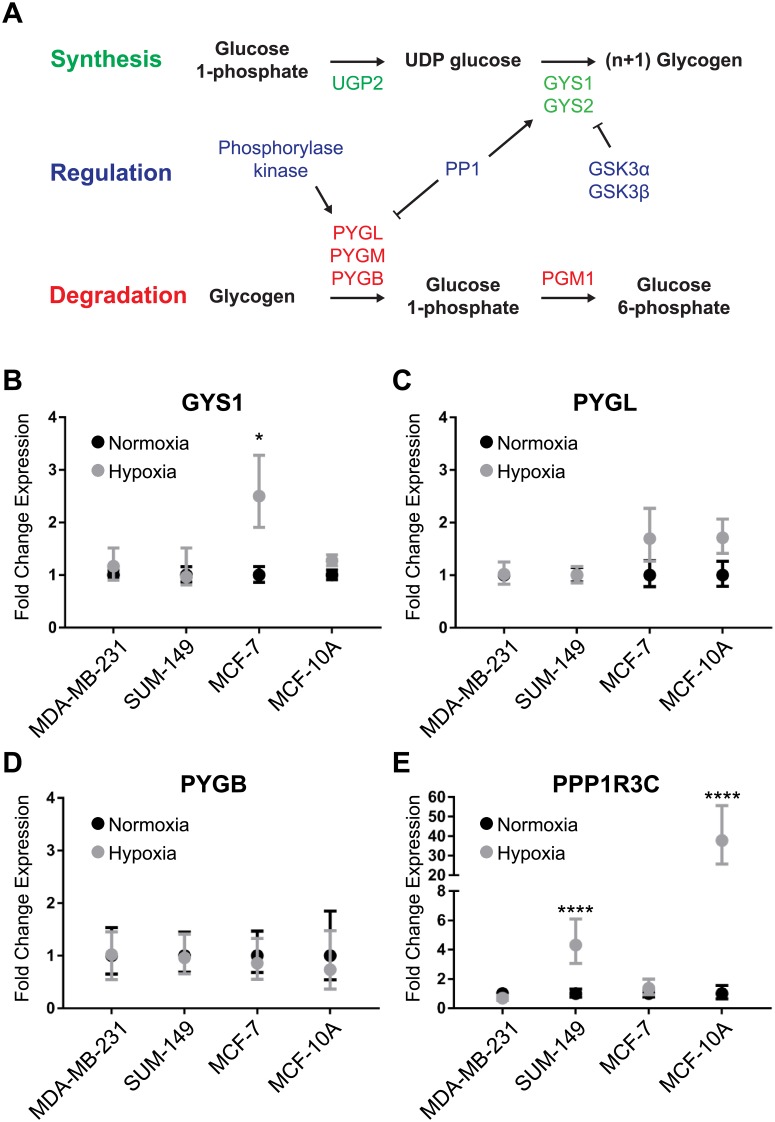
Glycogen pathway gene expression changes in breast cancer cells exposed to hypoxia. (A) Schematic of the glycogen synthesis and degradation pathways. (B-E) qPCR of (B) GYS1, (C) PYGL, (D) PYGB, and (E) PPP1R3C gene expression in MDA-MB-231, SUM-149, MCF-7, and MCF-10A cells. Points represent fold-change gene expression of cells exposed to hypoxia for 24h compared to normoxia for 24h. Normoxia values normalized to 1 are also displayed with associated error. Error bars represent SEM, n = 3. *p<0.05 ****p<0.0001.

We then investigated whether hypoxia-induced transcriptional changes in glycogen metabolism genes contribute to the observed glycogen accumulation under hypoxia. Glycogen synthesis and degradation pathways are outlined in Fig 2A. Glycogen synthase expression increased significantly under hypoxia in MCF-7 cells only (Fig 2B). We hypothesized that decreases in glycogen phosphorylase (PYGL/B) would also account for increased glycogen accumulation; however, no significant changes in expression were observed between normoxic and hypoxic conditions (Fig 2C and 2D) for PYGL/B. Expression of GYS2, GSK3α, GSK3β, PYGM, GYG1, and GYG2 were also evaluated showing no significant changes between normoxia and hypoxia (S2 Fig). However, there was a significant increase in the PP1 complex member PPP1R3C observed in SUM-149 and MCF-10A cells under hypoxic conditions (Fig 2E). These findings indicate that glycogen accumulation is likely not due to transcriptional regulation of the synthesis/degradation rate-limiting enzymes, but to other processes, such as allosteric regulation or post-translational modifications.

**Fig 3:**
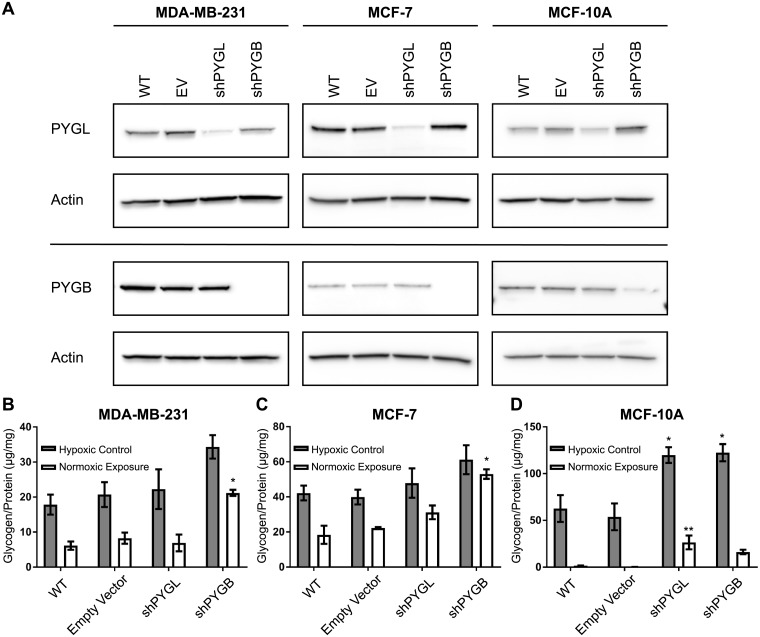
Glycogen phosphorylase brain isoform knockdown inhibits glycogen utilization in breast cancer cells. (A) Western blot of liver and brain glycogen phosphorylase (PYGL and PYGB) in wild-type, either empty vector, shPYGL and shPYGB transduced MDA-MB-231, MCF-7, and MCF-10A cells. Actin is shown as a loading control(B-D) Glycogen levels normalized to protein in lysate after 24h in hypoxia (Hypoxic Control) or 24h in hypoxia with subsequent 24h in normoxia (Normoxic Exposure) for wild-type, empty vector, shPYGL, and shPYGB in (B) MDA-MB-231, (C) MCF-7, and (D) MCF-10A cells, respectively. Error bars represent SEM of n = 3. *p<0.05, **p<0.01 compared to EV by multiple comparison one-way ANOVA.

We hypothesized that the ability to mobilize glucose from glycogen is an important vulnerability of aggressive breast cancers in that the degradation of glycogen is needed for invasion and motility. In order to observe whether the ability to store and utilize glycogen under hypoxia affects the ability of breast cancer cells to proliferate, migrate, and invade, cells that are unable to use glycogen stores are a necessary negative control. Since there were no consensus transcriptional changes observed that may account for glycogen accumulation under hypoxia, we knocked down glycogen phosphorylase, the rate-limiting enzyme of glycogen degradation in order to inhibit the breast cancer cells from utilizing glycogen. Breast cells express both the liver (PYGL) and brain (PYGB) isoforms of glycogen phosphorylase; therefore, we designed shRNA vectors against both PYGL and PYGB. We confirmed PYGL and PYGB knockdown in our cell models by western blot (Fig 3A) and qPCR (S3 Fig) compared to wild-type (WT) and empty-vector (EV) controls. To determine if glycogen utilization is regulated by PYGL or PYGB or both, we allowed the shPYGL and shPYGB cells to accumulate glycogen for 24hr in hypoxia (hypoxic control), then transferred the cells to normoxia for 24hr to deplete their glycogen stores (normoxic exposure), and subsequently tested their levels of glycogen (Fig 3B–3D).

Loss of PYGB but not PYGL in MDA-MB-231 (Fig 3B) and MCF-7 (Fig 3C) resulted in significantly more glycogen retention (p<0.05) under normoxia compared to wild-type and empty vector control cells. This indicates that PYGB, but not PYGL, is responsible for degradation of hypoxic glycogen stores in these cells. In normal-like breast epithelial MCF-10A cells, loss of PYGL resulted in significantly more glycogen retention under normoxia (p<0.01) and loss of either PYGL and PYGB maintained significantly more glycogen under hypoxia (p<0.05) compared to wild-type and empty vector controls. This indicates that both PYGL and PYGB are responsible for glycogen degradation in MCF-10A cells. The increase in glycogen stores under hypoxia observed in these cell lines is likely due to inhibition of degradation, rather than an increase in glycogen synthesis, given that we are reducing levels of glycogen phosphorylase, the canonical rate-limiting enzyme of glycogen degradation.

In addition, we generated stably expressing shPYGB and shPYGL vectors in SUM-149 cells. We confirmed the efficacy of PYGB or PYGL knockdown by RNA and protein expression (S4A and S4B Fig). In SUM-149, loss of PYGL, rather than PYGB, abrogated glycogen utilization (S4C Fig). Loss of PYGL in SUM-149 cells resulted in cessation of cellular propagation and, eventually, cell death. Future studies will focus on alternate methods of impeding glycogen utilization in SUM-149 cells in order to determine its effects on invasion and metastasis. Overall, these data show that the glycogen phosphorylase isoform responsible for the majority of glycogen degradation varies depending on breast cell line, with PYGB being the primary isoform responsible in MDA-MB-231 and MCF-7, and both PYGL and PYGB contributing in normal-like MCF-10A.

**Fig 4:**
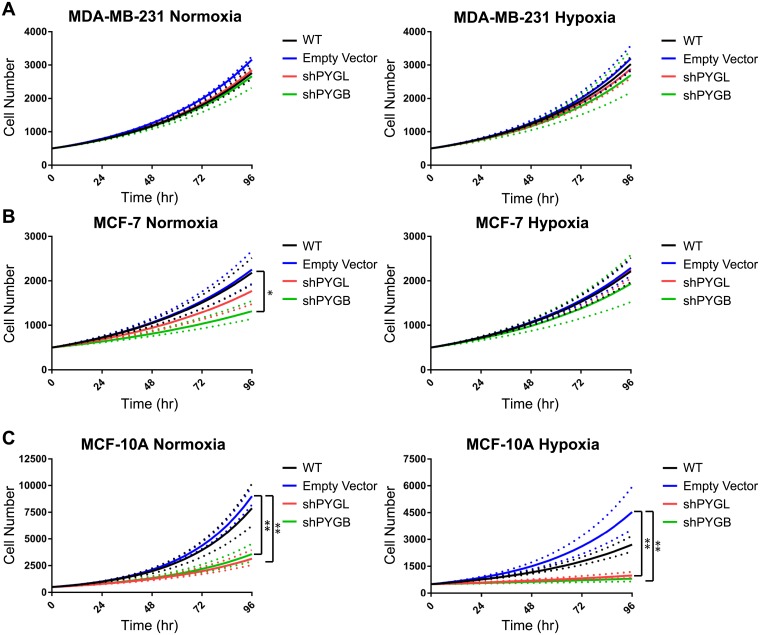
Loss of glycogen phosphorylase inhibits proliferation in MCF-7 and normal-like MCF-10A cells but not MDA-MB-231. Exponential curve fit of (A) MDA-MB-231, (B) MCF-7, and (C) MCF-10A cell proliferation in normoxic and hypoxic conditions, respectively. Dotted line indicates +/- standard error for n = 3. *p<0.05 **p<0.01 compared to EV by multiple-comparison one-way ANOVA on doubling times.

Proliferative effects due to the loss of either PYGL or PYGB were observed under normoxic and hypoxic conditions by live-cell bright-field imaging and cell counting every four hours for five days (Fig 4). Cell counts from the exponential growth phase and corresponding growth rate were determined. Doubling times for each cell line are reported in Table 1. Proliferation of shPYGL and shPYGB MDA-MB-231 cell lines did not differ from wild-type or empty vector controls in normoxic or hypoxic conditions (Fig 4A, Table 1). MCF-7 shPYGB cells had a significantly decreased proliferation rate (p<0.05) compared to both wild type and empty vector controls under normoxia; however, there were no significant differences between the shPYG cell lines and controls under hypoxic conditions (Fig 4B, Table 1). Loss of PYGB and PYGL significantly decreased the proliferation rate of MCF-10A cells compared to the empty vector control (p<0.01) in normoxic and hypoxic conditions (Fig 4C, Table 1). These data correlate with Fig 3C and 3D in that the loss of PYGB but not PYGL in MCF-7 inhibited glycogen utilization and slowed proliferation whereas loss of either enzyme in MCF-10A inhibited glycogen utilization and slowed proliferation compared to controls.

**Table 1:** Glycogen phosphorylase knockdown decreases doubling time of MCF-7 and MCF-10A cells.

Cell Line	Vector	Normoxia Mean Doubling Time days (+/- SD)	p-value compared to WT	p-value compared to EV	Hypoxia Mean Doubling Time days (+/- SD)	p-value compared to WT	p-value compared to EV
**MDA-MB-231**	WT	1.62 (1.04–1.56)	-	0.28	1.54 (1.48–1.60)	-	0.97
	EV	1.50 (1.48–1.53)	0.28	-	1.50 (1.41–1.59)	0.97	-
	shPYGL	1.60 (1.58–1.61)	0.97	0.47	1.58 (1.49–1.68)	0.98	0.83
	shPYGB	1.66 (1.52–1.81)	0.94	0.14	1.65 (1.44–1.88)	0.76	0.51
**MCF-7**	WT	1.88 (1.72–2.06)	-	0.997	1.86 (1.72–2.02)	-	0.99
	EV	1.84 (1.65–2.05)	0.997	-	1.82 (1.73–1.92)	0.99	-
	shPYGL	2.19 (1.88–2.56)	0.52	0.42	1.84 (1.73–1.96)	0.999	0.999
	shPYGB	**2.87 (2.45–3.35)**	**0.02**	**0.01**	2.05 (1.69–2.49)	0.73	0.60
**MCF-10A**	WT	1.01 (0.92–1.10)	-	0.93	1.64 (1.50–1.80)	-	0.75
	EV	0.96 (0.93–0.99)	0.93	-	1.26 (1.12–1.41)	0.75	-
	shPYGL	**1.51 (1.34–1.70)**	**0.004**	**0.002**	**4.09 (3.21–5.21)**	**0.04**	**0.009**
	shPYGB	**1.41 (1.26–1.58)**	**0.01**	**0.005**	**5.72 (3.18–10.3)**	**0.007**	**0.002**

p-values were calculated using multiple comparison one-way ANOVA on ln-transformed rate constants of the exponential curves

**Fig 5:**
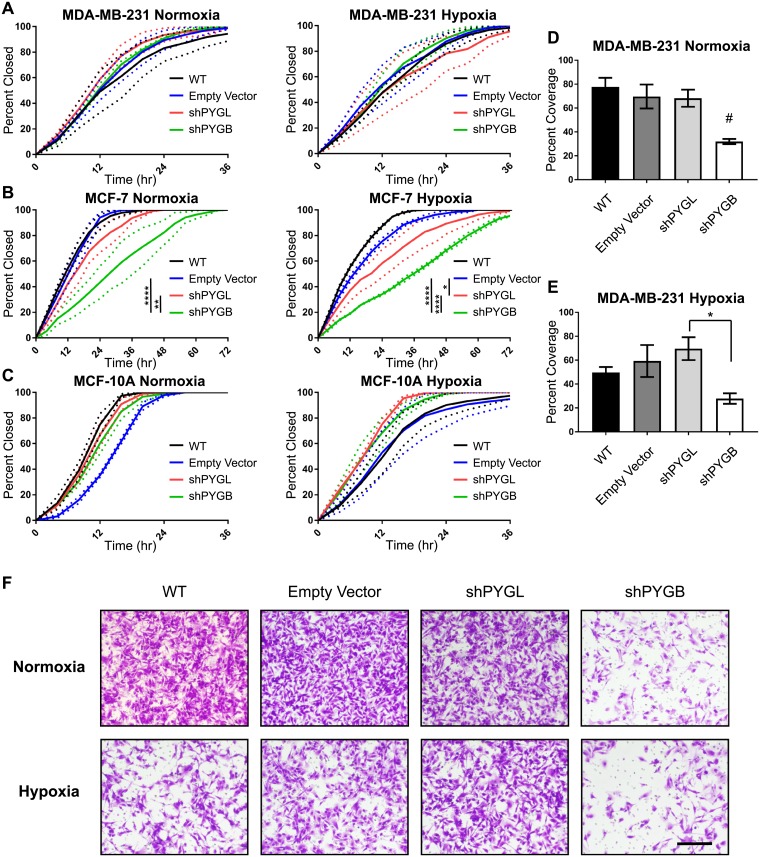
Loss of glycogen phosphorylase brain isoform inhibits wound-closure in MCF-7 and invasion in MDA-MB-231 cells. (A-C) Wound-healing assays for wild-type, empty vector, shPYGL, and shPYGB in (A) MDA-MB-231, (B) MCF-7, and (C) MCF-10A cells in normoxia and hypoxia, respectively. Data represented as percentage of wound closed based on 0hr time point image compared to images taken every 4hr. Dotted lines represent SEM at each timepoint of n = 3. *p<0.05, ****p<0.0001 compared to EV by two-way ANOVA. (D and E) Quantification of invasion assay of wild-type, empty vector, shPYGL, and shPYGB MDA-MB-231 cells in normoxia and hypoxia, respectively. Data represented as percent coverage of bottom side of transwell membrane after 24hr invasion from SFM to serum-containing media. Error bars represent SEM of n = 3 biological replicates. #p<0.05 compared to wild-type, empty vector, and shPYGL and *p<0.05 compared to shPYGL by multiple-comparison one-way ANOVA. (F) Representative 10X images of wild-type, empty vector, shPYGL, and shPYGB MDA-MB-231 invasion membranes in normoxia and hypoxia, scale bar = 200 μm.

Next, we determined whether the ability to utilize glycogen stores would also affect migration of breast cancer cells in both normoxic and hypoxic conditions using a wound-healing assay. Cells were plated in a 2-well insert which was removed after cells adhered to the bottom of the plate to create a uniform gap (“wound”) between the two groups of cells. Bright-field images were taken every four hours until the wound was fully closed, and the percentage of wound closure was calculated based on the area of the wound at each time point. Loss of PYGL and PYGB had no effect on wound-healing in MDA-MB-231 cells in normoxia or hypoxia (Fig 5A). In MCF-7 cells, loss of PYGB significantly inhibited wound-healing compared to wild-type (p<0.0001), empty vector control (p<0.0001), and to loss of PYGL (p<0.01), under normoxia (Fig 5B). Under hypoxic conditions, loss of PYGB again significantly inhibited wound closure compared to wild-type (p<0.0001), empty vector (p<0.0001), and loss of PYGL (p<0.0001) (Fig 5B). Loss of PYGL significantly inhibited wound closure of MCF-7 cells under hypoxic conditions compared to wild-type (p<0.0001) and empty vector (p<0.05) controls, but the effect was smaller from that seen for the shPYGB MCF-7 (Fig 5B). Even though knockdowns of PYGL and PYGB significantly reduced proliferation in MCF-10A, they had no effect on wound-healing of these normal-like epithelial cells in either normoxic or hypoxic conditions (Fig 5C).

Cellular invasion, as well as migration and proliferation, is an important phenotypic determinant of aggressive breast cancer progression and metastasis. TNBC cells MDA-MB-231 are highly invasive, unlike ER+ MCF-7 or normal-like MCF-10A. Using Matrigel-coated transwell inserts and serum as a chemoattractant, we determined that loss of PYGB, but not PYGL, significantly reduced the invasiveness (p<0.05) of MDA-MB-231 cells compared to wild-type and empty vector controls in normoxic conditions (Fig 5D). Under hypoxic conditions, loss of PYGB significantly reduced invasiveness compared to loss of PYGL (p<0.05) and trended towards reducing invasion compared to wild-type and empty vector controls, though not reaching statistical significance (Fig 5E). Representative images of invaded cells can be seen in Fig 5F. Importantly, these data are consistent with Fig 3B in that loss of PYGB, but not PYGL, inhibits glycogen utilization in MDA-MB-231.

## Discussion

It is well known that hypoxia increases migration, invasion, and metastasis in a variety of cancers, including breast cancers. Hypoxia also induces glycogen accumulation in cancer cells, promoting proliferation, protecting cells from reactive oxygen species, and preventing senescence [34,39,40]. Here we show that different types of breast cancer cells exhibit hypoxic glycogen accumulation and utilization of these glycogen stores contributes to proliferation, migration, and invasion.

All breast cancer cells tested increased glycogen stores in response to hypoxia. However, baseline normoxic glycogen levels and the amount of glycogen increase under hypoxic conditions varied widely between cell lines, with no discernible pattern based on receptor status or sub-type. Inflammatory breast cancer cells increased their glycogen stores in response to hypoxia by over 10-fold higher than other breast cancer cell types, suggesting that interventions based on inhibiting glycogen utilization may be more damaging to this aggressive breast cancer subtype. Overall, these data indicate that glycogen metabolism phenotypes in breast cancer may vary widely depending on each individual tumor. Even though there is no pattern to glycogen metabolism that is distinct for the commonly used breast cancer biomarkers, relative glycogen levels in patient biopsies can be determined by a simple and reliable histological test (PAS staining), thus potentially facilitating patient selection for interventions based on glycogen metabolism in the future.

We also found no single consensus glycogen gene expression signature that would account for the hypoxic glycogen accumulation observed in our breast cancer cells, indicating that there will be high degree of heterogeneity in the regulation of the common event we describe of glycogen storage under hypoxia. Previous studies have proposed that glycogen accumulation in breast cancer cells is due to HIF1α mediated increase in GYS1 and/or PPP1R3C expression in hypoxia [42,43]. In agreement with those prior results, we found an increase in GYS1 expression in MCF-7 cells and increased PPP1R3C expression in SUM-149 and normal-like MCF-10A cells; however, importantly, we find that there is no consensus glycogen-related gene expression among all breast cancer cells that leads to the observed hypoxic increase in glycogen. This result is important because it suggests that modulation of the rate limiting reactions of glycogen synthesis or degradation, rather than interventions on upstream targets, would have more general utility in breast cancer. This accumulation of glycogen could also be caused by allosteric regulation or phosphorylation/dephosphorylation of the rate-limiting enzymes of glycogen metabolism. Future studies will need to determine the exact mechanism of hypoxic glycogen accumulation based on glycogen synthase and glycogen phosphorylase regulation and the possible relation to HIF1α stabilization under hypoxia in breast cancer, in a context dependent manner.

Regardless of the mechanism of hypoxic glycogen accumulation, we successfully inhibited glycogen utilization in breast cancer using shRNA knockdown of the glycogen phosphorylase isoforms PYGL and PYGB. Previous work in the field focused solely on the liver isoform of glycogen phosphorylase [40]. However, we determined that the brain isoform of glycogen phosphorylase is primarily responsible for glycogen degradation in MDA-MB-231 and MCF-7 breast cancer cells and both isoforms contribute in normal-like MCF-10A cells. This novel finding should inform future glycogen metabolism studies in breast and other cancers to include all isoforms of glycogen phosphorylase in addition to the well-studied liver isoform.

Inhibition of glycogen utilization also led to drastic phenotypic changes in breast cancer cells in both hypoxic and normoxic conditions. Proliferation was reduced in shPYGB MCF-7 cells and both shPYGL and shPYGB normal-like MCF-10A cells, which matches with the inhibition of glycogen utilization seen in these cells. In the ER+ MCF-7 breast cancer cell line, wound-healing was also inhibited in the shPYGB cells. Wound-healing assays measure the ability of cells to move and grow outwards from an area of dense cell population. Without the ability to utilize glycogen, MCF-7 cells were unable to close the wound as efficiently as the control or the shPYGL cells. This effect was not seen in the non-cancerous, normal-like breast epithelial MCF-10A cells, indicating that glycogen usage to promote migration is a cancer-specific phenotype and thus a possible vulnerability. Additionally, in the TNBC MDA-MB-231 cells, inhibition of glycogen utilization by PYGB knockdown led to a significant decrease in invasive potential, reaffirming the importance of the brain isoform, PYGB, in advantaging cancer cells to more aggressive phenotypes based on enhanced glycogen availability.

While all breast cells tested increased glycogen storage under hypoxia, glycogen utilization as promoted by the brain isoform of glycogen phosphorylase, PYGB, affects migration and invasion phenotypes only in cancer cells and not in normal-like epithelial cells. These findings suggest PYGB as a potential novel target to reduce invasiveness and metastasis of breast cancers. Future work will focus on recapitulating these *in vitro* results in *in vivo* tumor xenograft and metastasis studies, as well as investigating the anti-metastatic effects of treatments with glycogen phosphorylases inhibitors, such as ingliforib [46].

## Supporting information

S1 Table Primers for qPCR of glycogen genes. (TIF)

S2 Table shRNA oligonucleotides for shPYGL and shPYGB. (TIF)

S1 Image Original Western blot images for MDA-MB-231 shPYG cells. Left column are chemiluminescent images without ladder, right column has bright-field ladder image superimposed onto chemiluminescent image. In each image, PYGL is on the upper left, PYGB on the upper right, and corresponding Actin loading controls are shown at the bottom.
(EPS)

S2 Image Original Western blot images for MCF-7 shPYG cells. Left column are chemiluminescent images without ladder, right column has bright-field ladder image superimposed onto chemiluminescent image. In each image, PYGL is on the upper left, PYGB on the upper right, and corresponding Actin loading controls are shown at the bottom.
(EPS)

S3 Image Original Western blot images for MCF-10A shPYG cells. Left column are chemiluminescent images without ladder, right column has bright-field ladder image superimposed onto chemiluminescent image. In each image, PYGL is on the upper left, PYGB on the upper right, and corresponding Actin loading controls are shown at the bottom.
(EPS)

S4 Image Original Western blot images for SUM-149 shPYG cells. Left column are chemiluminescent images without ladder, right column has bright-field ladder image superimposed onto chemiluminescent image. In each image, PYGL is on the upper left, PYGB on the upper right, and corresponding Actin loading controls are shown at the bottom.
(EPS)

S1 Fig Glycogen accumulates in additional breast cancer cells under hypoxic conditions. (A-C) Glycogen levels normalized to total protein lysate in MDA-MB-468, BT-549, and SUM-190 cell lines at 24 and 48h after media change and exposure to normoxia or 1% O_2_ (hypoxia) conditions. Error bars equal SEM of n = 3. *p<0.05, **p<0.01 by multiple comparisons one-way ANOVA. (D and E) PAS stain of (D) MDA-MB-231 and (E) SUM-149 cells after 48h exposure to normoxia or 1% O2 conditions. Images taken at 10X magnification. Inset is amylase digestion control. Scale bar = 50 μm.
(EPS)

S2 Fig Additional glycogen pathway gene expression changes in breast cancer cells exposed to hypoxia. (A-F) qPCR of GYS2, GYG1, GYG2, PYGM, GSK3α, and GSK3β gene expression in MDA-MB-231, SUM-149, MCF-7, and MCF-10A cells. Points represent fold-change gene expression of cells exposed to hypoxia for 24h compared to normoxia for 24h. Normoxia values normalized to 1 are displayed with associated error. Error bars represent SEM, n = 3.
(EPS)

S3 Fig shPYGL and shPYGB reduces glycogen phosphorylase mRNA expression. (A-C) qPCR confirmation of PYGL and PYGB knockdown in MDA-MB-231, MCF-7, and MCF-10A cells, respectively. Error bars represent SEM of n = 3. *p<0.05, **p<0.01, ***p<0.001, ****p<0.001 compared to EV by multiple comparisons one-way ANOVA.
(EPS)

S4 Fig Glycogen phosphorylase knockdown in SUM-149 cells. (A) Western blot of liver (PYGL) and brain glycogen phosphorylase (PYGB) in wild-type, empty vector transduced, shPYGL and shPYG with actin loading control in SUM-149 cells. (B) qPCR confirmation of PYGL and PYGB knockdown in SUM-149, n = 1. (C) Glycogen levels normalized to protein in lysate after 24h in hypoxia (Hypoxic Control) or 24h in hypoxia with subsequent 24h in normoxia (Normoxic Exposure) for wild-type, empty vector, shPYGL, and shPYGB in SUM-149, n = 1.
(EPS)
