# Supplementary figures and images for: Breast cancers utilize hypoxic glycogen stores via PYGB, the brain isoform of glycogen phosphorylase, to promote metastatic phenotypes
Source: PLoS One. 2019 Sep 19;14(9):e0220973. doi: 10.1371/journal.pone.0220973 (PMC6752868; doi:10.1371/journal.pone.0220973)

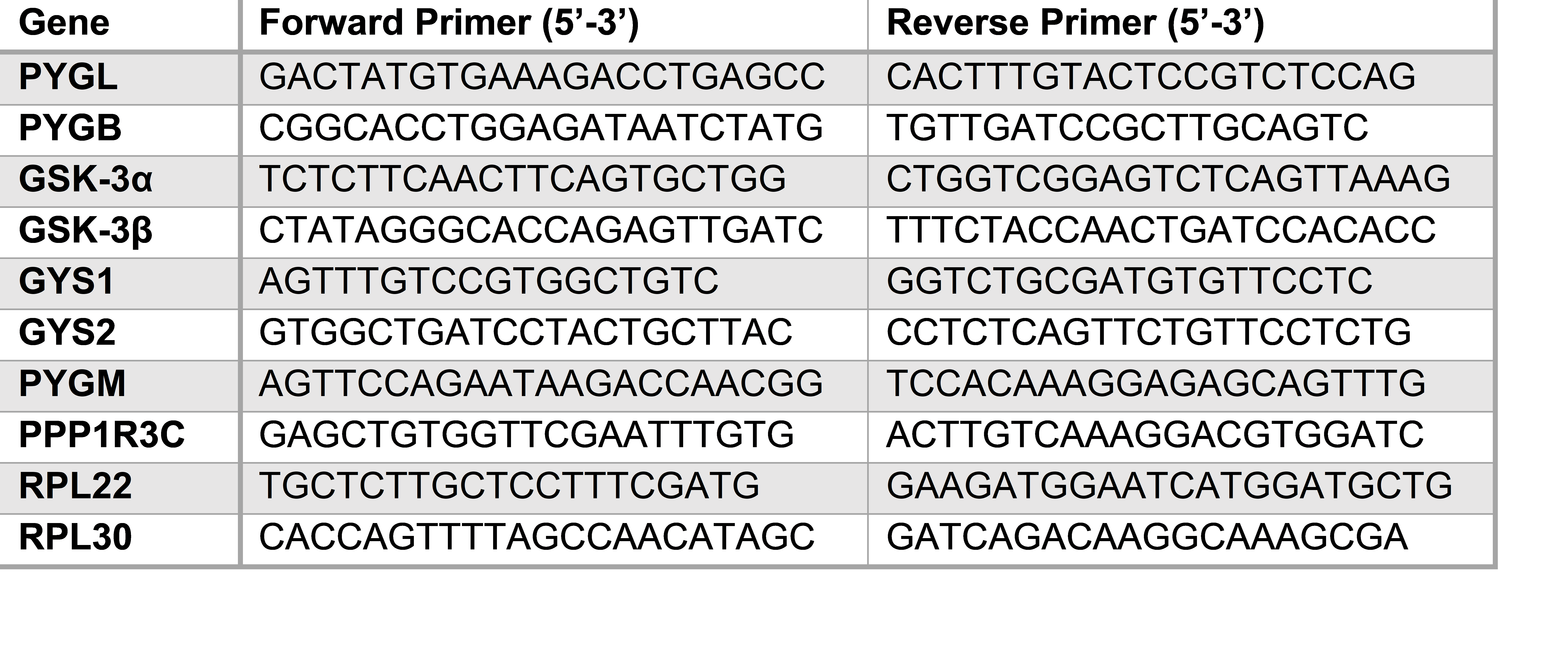

Supplement: S1 Table — (TIF) [file pone.0220973.s001.tif]

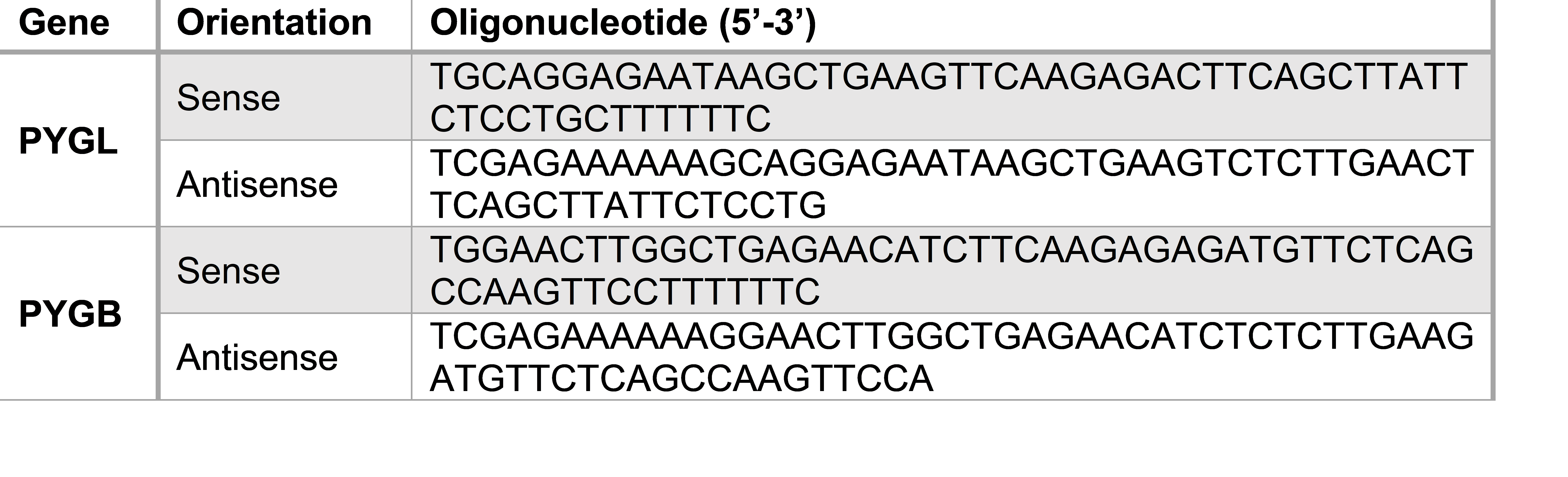

Supplement: S2 Table — (TIF) [file pone.0220973.s002.tif]
